# A New Paradigm in Eukaryotic Biology: HIV Tat and the Control of Transcriptional Elongation

**DOI:** 10.1371/journal.pbio.0030076

**Published:** 2005-02-15

**Authors:** Matjaz Barboric, B. Matija Peterlin

## Abstract

Studies of the transcriptional transactivator (Tat), a key regulatory protein of HIV, have yielded insight into the control of eukaryotic transcription

Viruses are intracellular pathogens that are subject to intense selective pressures during their ongoing battles within the host. To propagate successfully, they must exploit numerous machineries of the infected cell. Thus, studies of their replicative cycles have yielded fundamental insights into eukaryotic biology. A prime example is the human immunodeficiency virus (HIV), which is a lentivirus that causes the acquired immunodeficiency syndrome (AIDS). Unlike simpler oncoviruses that rely exclusively on host cell machinery, lentiviruses code for additional accessory and regulatory proteins that act as molecular switches at different stages of viral entry and exit from the infected cell. Studying the actions of these viral proteins has yielded understanding of diverse cellular functions such as the innate immunity against retroviruses, control of transcriptional elongation, export of macromolecules from the nucleus to the cytoplasm, and intracellular trafficking of proteins (reviewed in [1]).

The transcriptional transactivator (Tat) is a key regulatory protein of HIV. It is expressed early after the virus integrates into the cell, and stimulates the elongation of RNA polymerase II (RNAPII). This type of transcriptional control had not been previously appreciated; thus, work on Tat established a new paradigm in the field of eukaryotic biology. Moreover, these findings impacted greatly studies of cotranscriptional processing of nascent mRNA. To understand these processes better, we need to start with the basics of transcriptional control.

RNAPII is the enzyme that transcribes protein-coding genes in eukaryotic cells. Elegant studies in vitro first suggested that the simple recruitment of RNAPII to transcription units was not sufficient for the copying of genes and cotranscriptional processing of their transcripts. Rather, distinct steps could be defined, which began with the assembly of the preinitiation complex (PIC), promoter clearance, pausing, and arrest, and ended with efficient elongation of transcription (reviewed in [2]). The central component of PIC is the general transcription factor (GTF) TFIID, which contains the TATAbox- binding protein (TBP) and 12 to 15 TBP-associated factors (TAFs). TFIID acts as a “landing pad” for other GTFs and RNAPII to nucleate PIC assembly. Moreover, TAFs serve as coactivators to a diverse set of activators. Both an ordered stepwise assembly and the recruitment of the 100-plus-subunit “holoenzyme” have been proposed to be critical for the positioning of RNAPII at start sites of transcription.

Next, the GTF TFIIH unwinds the DNA, opens the transcription bubble, and phosphorylates serines at position 5 in the C-terminal domain (CTD) of the RPB1 subunit of RNAPII (reviewed in [2]). This phosphorylation is critical for the recruitment of complexes that put a 7-methylguanylate cap on the 5′ end of nascent transcripts. After the transcription complex clears the promoter, the negative transcription elongation factor (N-TEF) is recruited to the RNAPIIa (reviewed in [3]). It consists minimally of 5,6- dichloro-1-β-D-ribofuranosylbenzimidazole riboside (DRB)- sensitivity-inducing factor (DSIF) [4] and negative elongation factor (NELF) [5]. They bind and arrest RNAPII distal to the promoter cooperatively. Such arrested transcription complexes have now been found on many inducible genes in Drosophila melanogaster (reviewed in [6]) and humans [7].

The transition to robust elongation depends on the positive transcription elongation factor b (P-TEFb) (reviewed in [3]). P-TEFb contains the cyclin-dependent kinase 9 (CDK9) and one of four possible C-type cyclins. When recruited to stalled transcription complexes, P-TEFb phosphorylates serines at position 2 in the CTD [8], the Spt5 subunit of DSIF [9], and the RD subunit of NELF [10]. These modifications result in heavily phosphorylated RNAPII (RNAPIIo), the recruitment of the Elongator, which contains splicing and polyadenylation machineries, and the conversions of DSIF and NELF into elongation factors. RNAPIIo now copies the gene and directs the cotranscriptional processing, i.e., splicing and polyadenylation, of primary transcripts. Upon successful polyA addition, the CTD phosphatase FCP1 dephosphorylates RNAPIIo. RNAPIIa dissociates from DNA, and the transcription cycle starts all over again (reviewed in [2]).

Tat is unique among transcriptional activators in eukaryotic cells in that it functions via RNA rather than DNA promoter elements (Figure 1). It binds the transactivation response element (TAR) that forms a stable RNA stem loop at the 5′ end of all viral transcripts. Thus, Tat requires minimally the transcription of TAR before it can stimulate HIV transcription from the long terminal repeat (LTR). Indeed, in the absence of Tat, RNAPIIa clears the HIV LTR successfully but soon arrests, yielding predominantly short viral transcripts [11]. Tat binds the 5′ bulge in TAR via its arginine-rich motif from positions 49 to 57, where a central arginine (R52) is key for this interaction. However, this binding is not sufficient for Tat's function in vivo. Adjacent to the arginine-rich motif lie N-terminal core and cysteine-rich regions, which form the activation domain of the protein. This activation domain binds cyclin T1 (CycT1) from P-TEFb, whose partner is CDK9 [12]. As a consequence, P-TEFb and Tat bind TAR cooperatively. The final proof that P-TEFb is the cellular cofactor for Tat came from studies of HIV transcription in murine cells, where the introduction of the human CycT1 protein restores Tat function [12]. The same effect can be achieved by substituting just the tyrosine with the cysteine at position 261, such as are found in murine and human CycT1 proteins, respectively [13]. A paper in this issue of *PLoS Biology* suggests that Tat and P-TEFb can also recruit TAF-independent transcription complexes to the HIV LTR [14] (Figure 1). Possibly, this assembly reflects interactions between CycT1 and the unphosphorylated CTD of RNAPIIa [15].

**Figure 1:**
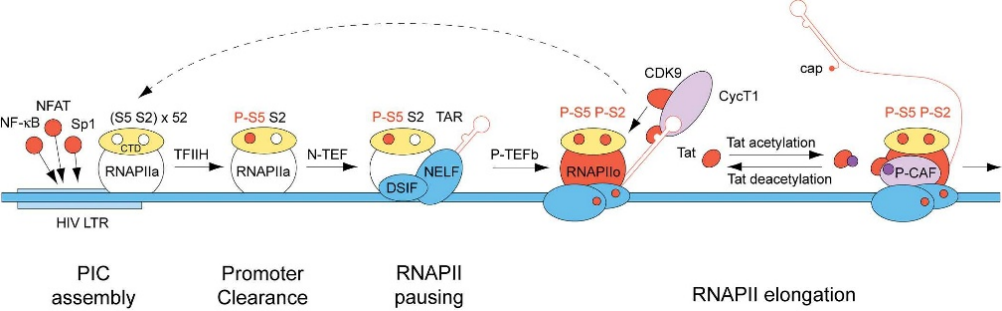
Activation of HIV Transcription by Tat Activators (red circles) that bind the HIV LTR promoter (light-blue rectangle) assemble the PIC and recruit RNAPIIa to the start site of transcription. For simplicity, only RNAPIIa in the PIC is presented. The yellow sphere with two open circles, depicting serines at position 5 and 2 within the CTD (S5 and S2, respectively), represents the unphosphorylated CTD of RNAPIIa (white sphere). TFIIH, which performs DNA-helicase and CTD-kinase activities, melts the DNA and phosphorylates S5 (red circle in the CTD; P-S5), resulting in promoter clearance. RNAPIIa transcribes TAR (red hairpin) and is paused by the binding of N-TEF, DSIF, and NELF, which are presented as blue spheres. The RD subunit of NELF binds the bottom stem in TAR. P-TEFb (comprising the red [CDK9] and pink [CycT1] spheres), which binds TAR together with Tat (small red sphere), phosphorylates S2 (red circle in the CTD; P-S2) to form elongating RNAPIIo (large red sphere). It also phosphorylates Spt5 in DSIF and RD in NELF, which become elongation factors, with the latter dissociating from TAR. In addition, P-TEFb, possibly independent of its kinase activity, assembles PIC via recruitment of TBP and RNAPIIa (dotted arrow). The phosphorylated CTD in RNAPIIo now binds the Elongator, which contains splicing machinery and polyadenylation factors. The red sphere at the 5′ end of the HIV transcript (red line) represents its cap. Finally, p300 acetylates Tat (magenta circle) and dissociates it from TAR. Acetylated Tat binds P-CAF and transfers it to RNAPIIo, possibly facilitating chromatin remodeling. Collectively, efficient RNAPII elongation of viral transcription ensues.

The assembly and disassembly of the complex between PTEFb, Tat, and TAR is a regulated process in vivo. Whereas the phosphorylation of CDK9 strengthens this complex [16], the acetylation of the lysine at position 50 in Tat weakens it [17]. Upon this disruption, acetylated Tat is liberated from P-TEFb and recruits the p300/CREB-binding protein– associated factor (P-CAF) to the elongating RNAPIIo, most likely facilitating chromatin remodeling. In this issue of PLoS Biology, Pagans et al. now demonstrate that acetylated Tat is deacetylated by SIRT1 [18] (Figure 1). In this way, Tat can reassemble with P-TEFb on TAR.

Clearly, P-TEFb plays a key role in the control of transcriptional elongation. Although Tat was the first activator known that could recruit P-TEFb to initiating RNAPII, additional members of this group were soon identified. They include the androgen receptor, c-Myc, the class II transactivator (CIITA), myoblast determination protein (MyoD), and nuclear factor κ-B (NF-κB). The last one is of great interest as it explains how the HIV genome can be transcribed before the synthesis of Tat [19]. Cellular activation triggers the nuclear translocation of NF-κB, where it binds the HIV enhancer, leading to the stimulation of viral transcription. It is not surprising that proviral latency, in which low levels of transcription or only short HIV transcripts containing TAR are observed, would in large part reflect the absence of these activators. Indeed, in many of these latently infected cells, the induction of NF-κB or the addition of Tat leads to the reactivation of viral replication and spreading of the infection [20,21].

Recently, important aspects of the regulation of P-TEFb have been revealed (Figure 2). Of interest, P-TEFb exists in two complexes in cells [22,23]. The larger measures approximately 500 kDa and contains the hexamethylene bisacetamide (HMBA)–induced protein 1 (HEXIM1) and 7SK small nuclear RNA (snRNA) in addition to P-TEFb [24,25]. In this large complex, Cdk9 is enzymatically inactive. HEXIM1 was identified as the inducible gene following the exposure of vascular smooth muscle cells to a potent differentiating agent, HMBA [26]. 7SK snRNA is one of the most abundant snRNA species, whose function remained a mystery for over a decade. Of interest, targeting of P-TEFb by HEXIM1 and 7SK snRNA contributes significantly to the control of cell growth and differentiation. For example, growth signals liberate P-TEFb from the large complex in the course of cardiac hypertrophy in mice, a disease characterized by the enlargement of myocytes due to a global increase in mRNA synthesis [27]. Also, following stress, ultraviolet light, or the administration of actinomycin D and DRB to cells, the large complex is converted to the small complex to stimulate transcription [22,23].

**Figure 2:**
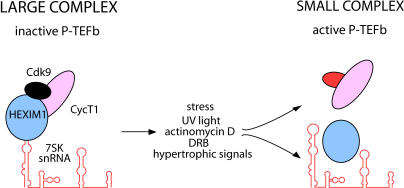
Inhibition of P-TEFb by the Coordinate Actions of HEXIM1 and 7SK snRNA HEXIM1 (blue sphere) binds the 5′ half of 7SK snRNA (red structure with multiple hairpins). Upon this binding, P-TEFb joins this RNA–protein complex and becomes enzymatically inactive, depicted by CDK9 as a black sphere. For simplicity, only the CDK9/CycT1 heterodimer is presented. Multiple stimuli, including stress, ultraviolet light, actinomycin D, DRB, and hypertrophic signals, dissociate HEXIM1 and 7SK snRNA from P-TEFb, possibly by preventing the RNA–protein interaction. In this way, P-TEFb is rendered active, depicted by CDK9 as a red sphere.

How central is P-TEFb to eukaryotic transcription? In Saccharomyces cerevisiae, there are two candidates for PTEFb, CTDK-1 and Bur1/2. CTDK1-negative but not Bur1/Bur2-negative yeasts still grow, albeit poorly and only on rich media (reviewed in [2]). In Caenorhabditis elegans, genetic inactivation of CDK9 or CycT1 and CycT2 resulted in the inhibition of all RNAPII transcription [8]. Moreover, in D. melanogaster, following heat shock, PTEFb is recruited upstream of activated promoters [28]. Although no murine knockouts of subunits of P-TEFb have been reported, DRB and flavopiridol, two ATP analogs that inhibit the kinase activity of CDK9, can inhibit nearly all transcription by RNAPII in human cells [29]. Indeed, as P-TEFb is a coactivator of potent activators that mediate effects of enhancers and can itself activate transcription when placed on sites distal to promoter elements [15], it might mediate many more signaling events than those of heat shock, ultraviolet light, stress, and hypertrophy. Conversely, the inhibition of P-TEFb could explain the mode of action of some transcriptional repressors. Indeed, the global transcriptional repressor PIE-1, the regulator of embryogenesis in C. elegans, binds the histidine-rich stretch in CycT1, thus decoying P-TEFb away from RNAPII and blocking the elongation of transcription [30].

These are exciting findings and suggest a plethora of future experiments, including the genetic inactivation of subunits of P-TEFb and isoforms of HEXIM1 in the mouse. Of special interest are questions as to where to place this mechanism of transcriptional regulation in the hierarchy of competing or complementary processes. What roles do different P-TEFb complexes play in the transcription of specific genes? How central will the regulation of P-TEFb be to cellular growth, proliferation, and differentiation, and what roles will it play in normal development and disease states? As to HIV, how can we use our knowledge of P-TEFb to slow down viral replication and/or to eliminate the state of proviral latency in the host? Obviously, we are only at the beginning of this journey, which promises to change radically our view of eukaryotic transcription.

## Abbreviations

AIDS: acquired immunodeficiency syndrome
CDK9: cyclindependent kinase 9
CIITA: class II transactivator
CTD: C-terminal domain
CycT1: cyclin T1
DRB: 5,6-dichloro-1-β-D-ribofuranosylbenzimidazole riboside
DSIF: 5,6-dichloro-1-β-D-ribofuranosylbenzimidazole riboside-sensitivity-inducing factor
GTF: general transcription factor
HEXIM1: hexamethylene-bisacetamideinduced protein 1
HIV: human immunodeficiency virus
HMBA: hexamethylene bisacetamide
LTR: long terminal repeat
NF-κB: nuclear factor κ-B
N-TEF: negative transcription elongation factor
P-CAF: p300/CREB-binding protein–associated factor
PIC: preinitiation complex
P-TEFb: positive transcription elongation factor b
RNAPII: RNA polymerase II
snRNA: small nuclear RNA
TAF: TATA-boxbinding protein–associated factors
TAR: transactivation response element
Tat: transcriptional transactivator
TBP: TATA-box-binding protein

## References

[pbio-0030076-b1] Freed EO (2004). HIV-1 and the host cell: An intimate association. Trends Microbiol.

[pbio-0030076-b2] Sims RJ, Belotserkovskaya R, Reinberg D (2004). Elongation by RNA polymerase II: The short and long of it. Genes Dev.

[pbio-0030076-b3] Price DH (2000). P-TEFb, a cyclin-dependent kinase controlling elongation by RNA polymerase II. Mol Cell Biol.

[pbio-0030076-b4] Wada T, Takagi T, Yamaguchi Y, Ferdous A, Imai T (1998). DSIF, a novel transcription elongation factor that regulates RNA polymerase II processivity, is composed of human Spt4 and Spt5 homologs. Genes Dev.

[pbio-0030076-b5] Yamaguchi Y, Takagi T, Wada T, Yano K, Furuya A (1999). NELF, a multisubunit complex containing RD, cooperates with DSIF to repress RNA polymerase II elongation. Cell.

[pbio-0030076-b6] Lis J (1998). Promoter-associated pausing in promoter architecture and postinitiation transcriptional regulation. Cold Spring Harb Symp Quant Biol.

[pbio-0030076-b7] Sawado T, Halow J, Bender MA, Groudine M (2003). The β-globin locus control region (LCR) functions primarily by enhancing the transition from transcription initiation to elongation. Genes Dev.

[pbio-0030076-b8] Shim EY, Walker AK, Shi Y, Blackwell TK (2002). CDK-9/cyclin T (P-TEFb) is required in two postinitiation pathways for transcription in the C. elegans embryo. Genes Dev.

[pbio-0030076-b9] Ivanov D, Kwak YT, Guo J, Gaynor RB (2000). Domains in the SPT5 protein that modulate its transcriptional regulatory properties. Mol Cell Biol.

[pbio-0030076-b10] Fujinaga K, Irwin D, Huang Y, Taube R, Kurosu T (2004). Dynamics of human immunodeficiency virus transcription: P-TEFb phosphorylates RD and dissociates negative effectors from the transactivation response element. Mol Cell Biol.

[pbio-0030076-b11] Kao SY, Calman AF, Luciw PA, Peterlin BM (1987). Anti-termination of transcription within the long terminal repeat of HIV-1 by tat gene product. Nature.

[pbio-0030076-b12] Wei P, Garber ME, Fang SM, Fischer WH, Jones KA (1998). A novel CDK9- associated C-type cyclin interacts directly with HIV-1 Tat and mediates its high-affinity, loop-specific binding to TAR RNA. Cell.

[pbio-0030076-b13] Garber ME, Wei P, KewalRamani VN, Mayall TP, Herrmann CH (1998). The interaction between HIV-1 Tat and human cyclin T1 requires zinc and a critical cysteine residue that is not conserved in the murine CycT1 protein. Genes Dev.

[pbio-0030076-b14] Raha T, Cheng SWG, Green MR (2005). HIV-1 tat stimulates transcription complex assembly through recruitment of TBP in the absence of TAFs. PLoS Biol.

[pbio-0030076-b15] Taube R, Lin X, Irwin D, Fujinaga K, Peterlin BM (2002). Interaction between P-TEFb and the C-terminal domain of RNA polymerase II activates transcriptional elongation from sites upstream or downstream of target genes. Mol Cell Biol.

[pbio-0030076-b16] Garber ME, Mayall TP, Suess EM, Meisenhelder J, Thompson NE (2000). CDK9 autophosphorylation regulates high-affinity binding of the human immunodeficiency virus type 1 tat-P-TEFb complex to TAR RNA. Mol Cell Biol.

[pbio-0030076-b17] Kiernan RE, Vanhulle C, Schiltz L, Adam E, Xiao H (1999). HIV-1 tat transcriptional activity is regulated by acetylation. EMBO J.

[pbio-0030076-b18] Pagans S, Pedal A, North BJ, Kaehlcke K, Marshall BL (2005). SIRT1 regulates HIV transcription via Tat deacetylation. PLoS Biol.

[pbio-0030076-b19] Barboric M, Nissen RM, Kanazawa S, Jabrane-Ferrat N, Peterlin BM (2001). NF-kappaB binds P-TEFb to stimulate transcriptional elongation by RNA polymerase II. Mol Cell.

[pbio-0030076-b20] Brooks DG, Hamer DH, Arlen PA, Gao L, Bristol G (2003). Molecular characterization, reactivation, and depletion of latent HIV. Immunity.

[pbio-0030076-b21] Lin X, Irwin D, Kanazawa S, Huang L, Romeo J (2003). Transcriptional profiles of latent human immunodeficiency virus in infected individuals: Effects of Tat on the host and reservoir. J Virol.

[pbio-0030076-b22] Nguyen VT, Kiss T, Michels AA, Bensaude O (2001). 7SK small nuclear RNA binds to and inhibits the activity of CDK9/cyclin T complexes. Nature.

[pbio-0030076-b23] Yang Z, Zhu Q, Luo K, Zhou Q (2001). The 7SK small nuclear RNA inhibits the CDK9/cyclin T1 kinase to control transcription. Nature.

[pbio-0030076-b24] Michels AA, Nguyen VT, Fraldi A, Labas V, Edwards M (2003). MAQ1 and 7SK RNA interact with CDK9/cyclin T complexes in a transcriptiondependent manner. Mol Cell Biol.

[pbio-0030076-b25] Yik JH, Chen R, Nishimura R, Jennings JL, Link AJ (2003). Inhibition of P-TEFb (CDK9/Cyclin T) kinase and RNA polymerase II transcription by the coordinated actions of HEXIM1 and 7SK snRNA. Mol Cell.

[pbio-0030076-b26] Kusuhara M, Nagasaki K, Kimura K, Maass N, Manabe T (1999). Cloning of hexamethylene-bis-acetimide-inducible transcript, HEXIM1, in human vascular smooth muscle cells. Biomed Res.

[pbio-0030076-b27] Sano M, Abdellatif M, Oh H, Xie M, Bagella L (2002). Activation and function of cyclin T-Cdk9 (positive transcription elongation factor-b) in cardiac muscle-cell hypertrophy. Nat Med.

[pbio-0030076-b28] Lis JT, Mason P, Peng J, Price DH, Werner J (2000). P-TEFb kinase recruitment and function at heat shock loci. Genes Dev.

[pbio-0030076-b29] Chao SH, Price DH (2001). Flavopiridol inactivates P-TEFb and blocks most RNA polymerase II transcription in vivo. J Biol Chem.

[pbio-0030076-b30] Zhang F, Barboric M, Blackwell TK, Peterlin BM (2003). A model of repression: CTD analogs and PIE-1 inhibit transcriptional elongation by PTEFb. Genes Dev.

